# Profile analysis of hepatic porcine and murine brain tissue slices obtained with a vibratome

**DOI:** 10.7717/peerj.932

**Published:** 2015-04-30

**Authors:** G Mattei, I Cristiani, C Magliaro, A Ahluwalia

**Affiliations:** 1 Research Center “E. Piaggio,” University of Pisa, Pisa, Italy; 2 Institute of Clinical Physiology, National Research Council, Pisa, Italy

**Keywords:** Brain, Vibratome, Liver, Precision-cut slices, Graphical user interface

## Abstract

This study is aimed at characterizing soft tissue slices using a vibratome. In particular, the effect of two sectioning parameters (i.e., step size and sectioning speed) on resultant slice thickness was investigated for fresh porcine liver as well as for paraformaldehyde-fixed (PFA-fixed) and fresh murine brain. A simple framework for embedding, sectioning and imaging the slices was established to derive their thickness, which was evaluated through a purposely developed graphical user interface. Sectioning speed and step size had little effect on the thickness of fresh liver slices. Conversely, the thickness of PFA-fixed murine brain slices was found to be dependent on the step size, but not on the sectioning speed. In view of these results, fresh brain tissue was sliced varying the step size only, which was found to have a significant effect on resultant slice thickness. Although precision-cut slices (i.e., with regular thickness) were obtained for all the tissues, slice accuracy (defined as the match between the nominal step size chosen and the actual slice thickness obtained) was found to increase with tissue stiffness from fresh liver to PFA-fixed brain. This quantitative investigation can be very helpful for establishing the most suitable slicing setup for a given tissue.

## Introduction

Vibrating blade microtomes or vibratomes are commonly used for obtaining precision-cut slices from soft fresh tissues. Unlike classical sectioning procedures based on the use of microtomes, vibratome slicing does not require any tissue fixation, dehydration and embedding, thus cell viability and native tissue structure are conserved. Fresh tissue slices are suitable candidates for *in vitro* tissue models (Parrish, Gandolfi & Brendel, 1995; Parrish et al., 2002; Van de Bovenkamp et al., 2005; Groothuis & de Graaf, 2013) as well as for structural and morphometric analysis (Karim et al., 2013; Eide et al., 2014). For instance, precision-cut liver slices are powerful tools for the *in vitro* study of pharmacological metabolism, toxicology and efficacy of novel substances under standardized conditions (Van de Bovenkamp et al., 2005; Van de Bovenkamp et al., 2006; Van de Bovenkamp et al., 2007; Karim et al., 2013; Eide et al., 2014). They have been used extensively for rank-ordering the toxicity of chemicals and examining the mechanisms of liver injury as well as for investigating the induction of cytochrome P-450 enzymes and the expression of stress proteins or peroxisomal enzymes, thus offering a valuable bridge between *in vivo* and cell culture systems (Gandolfi, Wijeweera & Brendel, 1996; Olinga & Schuppan, 2013). Notably, as the metabolic functions of pig liver are very similar to human liver, porcine hepatic tissue slices are often employed for *in vitro* model applications and bio-artificial liver devices (Vilei et al., 2001; Swindle, 2007). Brain tissue slices, on the other hand, are attractive for the evaluation of different morphometric features such as the total extent of dendrites and the number of branching points, as well as for 3D tissue reconstruction and analysis of neurons (Jin et al., 2003; Billeci et al., 2013; Golovyashkina et al., 2014).

Despite the widespread use of vibratomes for obtaining live tissue sections, it is difficult to find standard protocols or consolidated methods to determine sectioning parameters for generating precision cut slices with a desired thickness for a given application. This is partly because the selection of sectioning parameters is likely to depend widely on tissue type and donor (Zimmermann et al., 2009) and sample status (e.g., hydration Haji Maghsoudi, Hosseini Sharifabad & Karimzadeh, 2008; Png et al., 2008), and also because the sample embedding and processing for thickness analysis varies from report to report (i.e., Haji Maghsoudi, Hosseini Sharifabad & Karimzadeh, 2008; Zimmermann et al., 2009). Indeed there is no unique consensus on the correlation between the nominal and experimental thickness of vibratome slices. Most studies evaluate the experimental thickness of vibratome-sliced tissues through paraffin embedding procedures and generally report a shrinkage with respect to the nominal one, such as in Haji Maghsoudi, Hosseini Sharifabad & Karimzadeh (2008) and Christensen et al. (2007). In the latter study, the authors report a tissue shrinkage of 54% in the *z*-axis after sectioning and suggest that vibratome sections should typically be cut at 70–100 µm due to this substantial collapse (Christensen et al., 2007). This shrinking is most likely a result of sample dehydration during paraffin embedding, as discussed in Zimmermann et al. (2009), and is thus poorly representative of the experimental slice thickness after vibratome sectioning. Moreover, it is not obvious to always expect tissue shrinking after vibratome slicing. Indeed, Zimmerman and colleagues found the experimental thicknesses of porcine and bovine liver slices to be higher than the nominal vibratome step size used (i.e., 200 µm), even though they used paraffin embedding and subsequent cross sectioning to estimate slice thickness. These rather inconsistent findings motivated us to establish an experimental framework for the rapid generation and characterization of precision cut tissue slices without having to resort to lengthy trial and error experiments and tissue wastage. In particular, our main objective was to determine the “actual” slice thickness in the fully hydrated state after sectioning and subsequent equilibration in phosphate buffered saline (PBS). Notably, the “actual” thickness as defined here accounts for any deformation or swelling after sectioning and is representative of that of the slice during culture (e.g., for application to organotypic cultures for toxicity testing). In the present study, tissue slices were obtained from fresh porcine liver and from both fresh and paraformaldehyde-fixed (PFA-fixed) murine brain by varying two main vibratome sectioning parameters, i.e., the step size and the sectioning speed. These three different tissues were selected in order to have three samples differing in stiffness to evaluate the effect of varying vibratome slicing parameters on the actual slice thickness (i.e., after sectioning and equilibration). In particular, tissue stiffness increases from fresh pig liver to PFA-fixed brain. In a recent study we showed that the compressive modulus of fresh porcine liver samples harvested from one-year-old healthy pigs (as used in this work, Section ‘Sample preparation and slicing setup’) is ∼1.5 kPa (Mattei et al., 2014a). Aurand et al. (2014) reported that the compressive modulus for murine brain samples harvested from 3 months mice (as used in this study, Section ‘Sample preparation and slicing setup’) is ∼3.5 kPa. The stiffness of biological tissues is known to increase after PFA-fixation (Braet et al., 1998; Hutter et al., 2005; Franke et al., 2007), being about 20 times higher than that of fresh tissue for brain samples obtained by perfusing 4% PFA through the animal’s left ventricle (as in this work, Section ‘Sample preparation and slicing setup’) (Lee, 2011).

The other vibratome sectioning parameters (i.e., oscillation amplitude and blade angle) were kept constant as they are less crucial to the determination of slice thickness. In order to derive meaningful results with minimal artefacts due to tissue shrinkage, the actual slice thicknesses were evaluated by embedding fully hydrated vibratome-sliced samples in agarose gels. Unlike classical paraffin inclusion, agarose embedding does not require any tissue fixation and dehydration, which are likely to affect quantitative measurements of resultant slice thickness, as discussed above. The technique is widely used for processing delicate samples and is known to maintain their morphology intact (Wu, Baskin & Gallagher, 2012; Ke, Fujimoto & Imai, 2013). Agarose-embedded slices were sectioned perpendicularly to their surface obtaining transverse sections. The sections were imaged with an optical microscope and analyzed with a purposely-developed Graphical User Interface (GUI) to evaluate the actual slice thickness.

## Materials and methods

### Sample preparation and slicing setup

Fresh porcine hepatic tissue was collected from *n* = 2 one-year-old healthy pigs and cut into 1.5 × 0.5 × 0.5 cm^3^ samples, avoiding the Glisson’s capsule and macroscopic vasculature. The tissue was obtained from a local abattoir, as a slaughter by-product. Fresh murine brains were collected from *n* = 2 three-month-old mice which were deeply anesthetized by intraperitoneal injection of chloral hydrate (400 mg/kg) and then perfused through the left ventricle with 50 mL of 10 mM phosphate buffered saline (PBS 1×; Sigma-Aldrich, Milan, Italy). PFA-fixed brains were obtained from *n* = 2 three-month-old mice treated as described for fresh tissue and then perfused with 200 mL of 4% paraformaldehyde (PFA, pH 7.4) fixative solution prepared in 0.1 M PBS (Sigma-Aldrich, Milan, Italy). Murine brains were cut along to their sagittal plane, obtaining two samples. Mouse perfusion was performed at the Department of Translational Research New Technologies in Medicine and Surgery of the University of Pisa. Experiments were conducted in conformity with the European Communities Council Directive of 24 November 1986 (86/609/EEC and 2010/63/UE) and in agreement with the Italian DM26/14. Experiments were approved by the Italian Ministry of Health and Ethical Committee of the University of Pisa.

A Leica VT1200 S vibratome (Leica Microsystems, Nussloch, Germany) was used to obtain tissue slices. Each sample was fixed with superglue onto specimen plates and then cut using a stainless steel razor blade (Gillette, Milan, Italy), under buffered conditions with ice-cold PBS 1×. Block advance calibrations were used to set up and calibrate the vibratome device. In particular, 10 slices of 200 µm thickness were cut from a block of pig liver. Block thickness was measured before and after cutting with a caliper, averaging measurements from 4 different points over the surface of the block. The results were within 5% of the expected 2 mm. The following cutting settings were used: blade angle, 18°; oscillation amplitude, 3 mm for liver, 1.5 mm for brain; sectioning speed, 0.1, 0.2 and 0.4 mm/s for liver, 0.05 and 0.2 mm/s for PFA-fixed brain; step size, 100, 200 and 400 µm. In the case of fresh brain, only one sectioning speed (0.2 mm/s) was used on the basis of the results obtained from fixed tissue.

### Thickness evaluation

**Figure 1:**
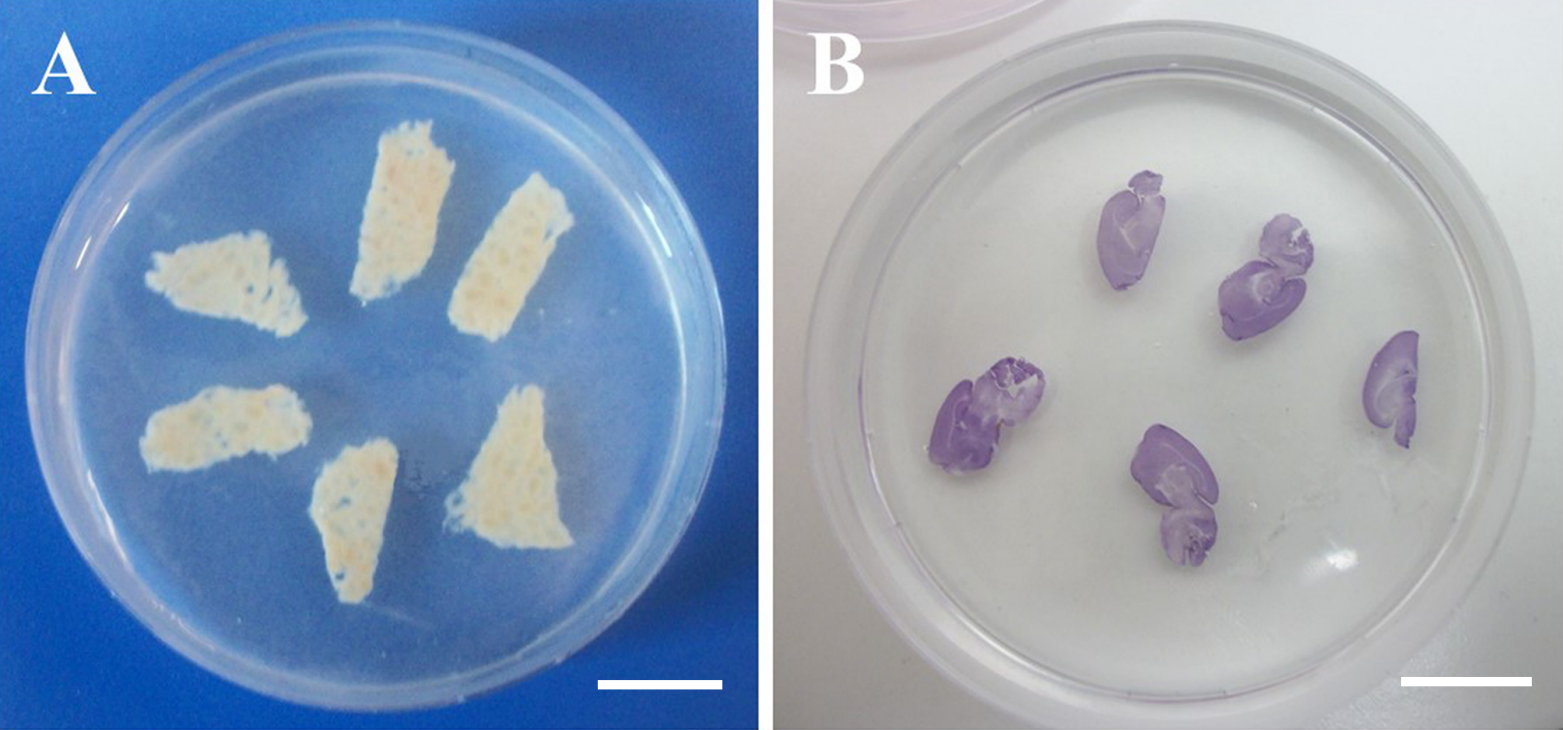
Agarose-embedded tissue slices. (A) Fresh porcine liver. (B) Haematoxylin-stained PFA-fixed murine brain. Scale bars: 1 cm.

After cutting, the slices were equilibrated in PBS 1× and then embedded in a 1% w/v agarose gel (A9539; Sigma-Aldrich, Milan, Italy) prepared in deionized water (Fig. 1). The slice-containing agarose gel, which was formed at room temperature via thermal gelation, allowed easy and quick embedding of hydrated slices. As discussed in the introduction, this procedure enables the evaluation of slice thickness in the hydrated state with minimal distortion. To enhance contrast, brain tissue slices were stained with haematoxylin (Sigma-Aldrich, Milan, Italy) prior to embedding (Fig. 1B).

Agarose-embedded slices were cut perpendicularly to their surface using a guillotine-like custom slicer equipped with a microtome blade. The cross-sections were immediately placed onto a glass slide and imaged with an Olympus IX81 optical microscope (Olympus, Milan, Italy) at 1.25× magnification. Acquired images were processed with a purposely-developed software implemented in Matlab^®^ (The Mathworks Inc., Natick, MA, USA), named STEGUN (after the mathematician Irene Stegun, the name also stands for “Slice Thickness Evaluation GUi for Non-expert users”). The software is available for download at http://www.centropiaggio.unipi.it/software.

**Figure 2:**
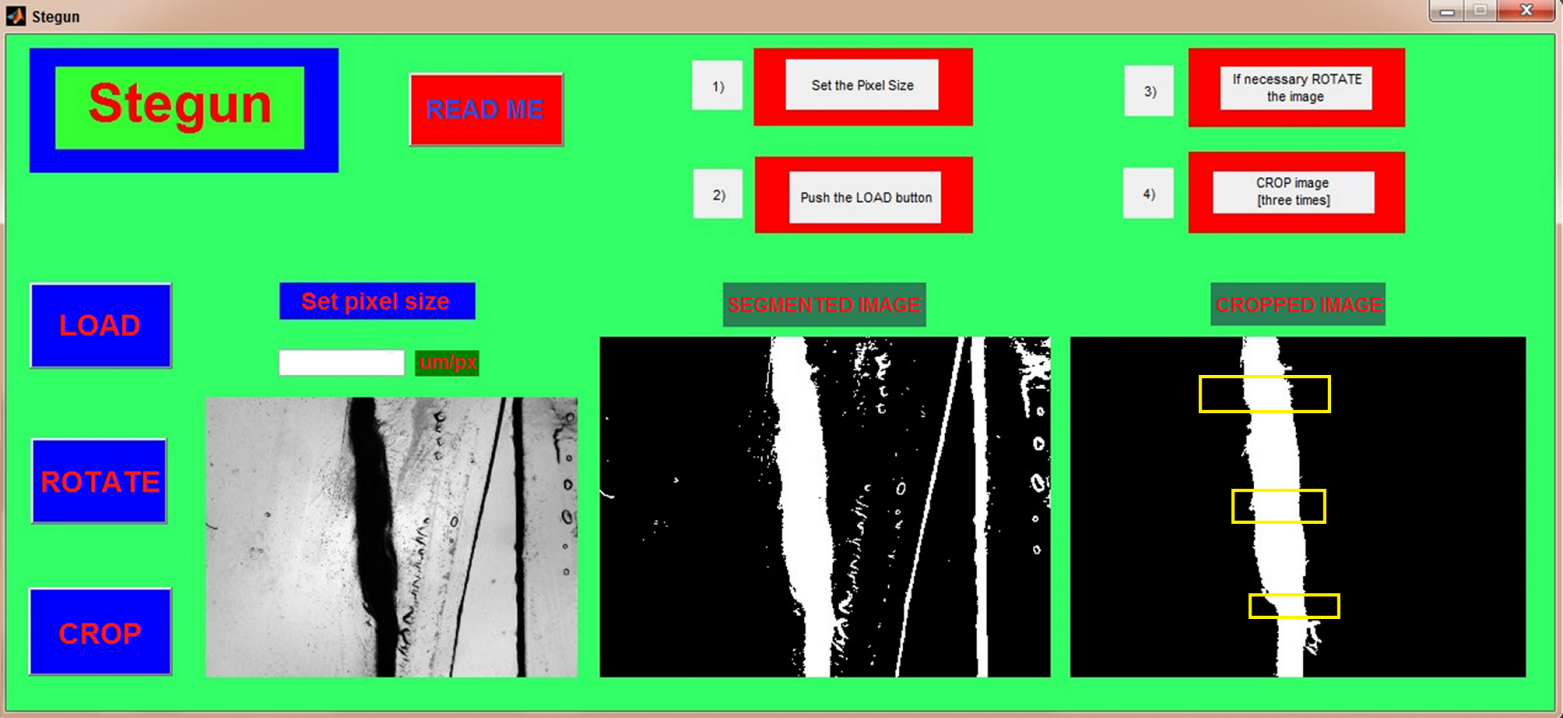
STEGUN: a semi-automated tool to evaluate slice thickness. After launching the GUI, the user has to set the image pixel size by typing its value (in µm/pixel) within the dedicated box (white rectangle in the left part of the GUI) and load the image to analyze using the LOAD button. The original image appears in the leftmost panel and its corresponding binarized version in the central one. At this point the user has to select the slice by dragging the mouse over the region of interest. STEGUN then returns the cropped image in the rightmost panel. If necessary, the user can rotate the cropped image to vertically align the slice by clicking on the ROTATE button: image rotation is defined by manually selecting the two opposite extremities that identify the principal axis of the slice using the mouse cursor. Finally, the user must select three different rectangular segments of the cropped image in the rightmost panel by clicking and dragging the mouse. The three yellow rectangles shown in rightmost panel in the GUI represent an example of three possible segments over which the thickness is calculated. As described in section ‘Thickness evaluation’, the results are returned to the user as mean thickness ± standard deviation.

STEGUN’s simple GUI allows a semi-automated evaluation of slice thickness in 4 simple steps, as illustrated in Fig. 2. Briefly, after setting the pixel size and loading the image, the latter is binarized through a thresholding algorithm, then the pixel values are inverted to obtain a white object representing the slice (pixel level = 1) in a black background (pixel level = 0). To evaluate the slice thickness, the user has to select three rectangular segments including the slice from the processed binary image. For each of these three crops, the slice thickness is automatically evaluated by summing the pixel values and normalizing the result by the number of pixel rows of the crop. Finally, the computed result is multiplied by the pixel size to obtain the slice thickness in microns. STEGUN stores all the data in a data matrix and displays the result as the mean value ± standard deviation. In case of highly irregular slices (i.e., when the coefficient of variation, calculated as the ratio of the standard deviation to the mean value, is greater than 0.25) a warning message is returned to the user.

### Data analysis

At least 6 independent tissue slices were analyzed for each step size and sectioning speed investigated. Results are reported as the mean ± standard deviation, unless otherwise noted. For both porcine liver and PFA-fixed murine brain, the statistical significance between slice thicknesses obtained varying two different sectioning parameters (i.e., step size and sectioning speed) was analyzed using two-way ANOVA. Slice thickness for fresh murine brain was analyzed with one-way ANOVA, since only the slicing step size was varied, keeping the sectioning speed at a constant value. Post-hoc multiple comparisons between different groups of data were carried out using the Tukey’s honestly significant difference test (Tukey’s HSD test). Statistical analysis was implemented in OriginPro 9.0 (OriginLab Corporation, Northampton, Massachusetts, USA). Differences were considered significant at *p* < 0.05. In addition, to quantify the average mismatch between experimental and theoretical (nominal) slice thickness for a given tissue, all the step sizes and speeds investigated were considered to obtain an overall thickness change. In particular, for each of the step size-sectioning speed combinations the thickness change was calculated as (average measured thickness–step size)/step size and expressed as a percentage. The values thus obtained were averaged to get the overall thickness change for the tissue. Since only 2 animals were used per experiment, increasing the number of animals investigated may improve the statistical accuracy and robustness of the results presented.

## Results and Discussion

### Fresh porcine liver

**Figure 3:**
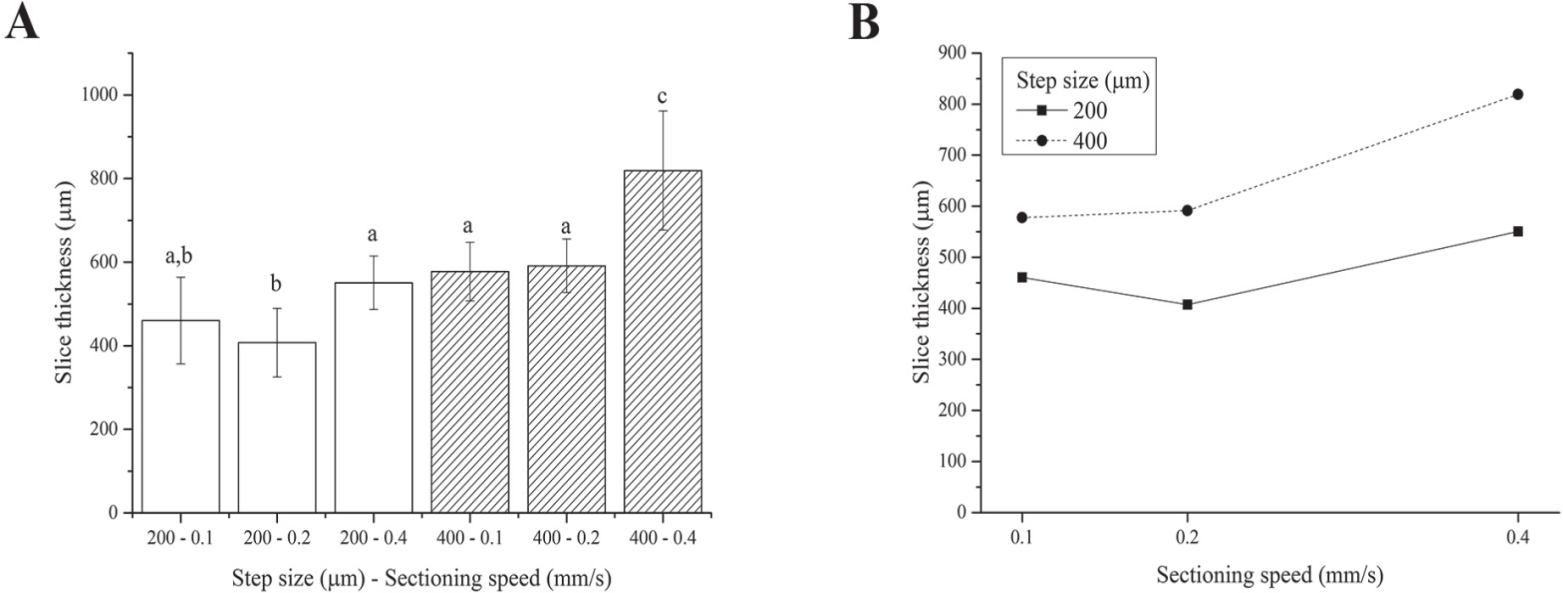
Fresh liver slice thicknesses. (A) Bar plot: different letters indicate significant differences between samples (*p* < 0.05). Different bar fillings indicate different step sizes. (B) Two-way ANOVA interaction plot: the interaction between the step size and the sectioning speed is not significant. Error bars represent standard deviations.

We were unable to obtain slices using the 100 µm step size because the blade tended to deform and scrape over the tissue rather than cut it, regardless of the sectioning speed. This is likely due to the very labile and floppy nature of fresh liver (Mattei et al., 2014b). Although the two-way ANOVA analysis showed that both the step size and sectioning speed have a significant effect on the resultant thickness of fresh liver slices, 4 of the 6 step size-sectioning speed combinations investigated (specifically 200-0.1, 200-0.4, 400-0.1 and 400-0.2 µm-mm/s) yielded similar slice thicknesses, with an average value of 540 ± 91 µm (Fig. 3A). Moreover, the interaction between the two factors (i.e., step size and sectioning speed) was not found to be significant: lines in Fig. 3B exhibit the same trend versus the sectioning speed, regardless of the step size.

Overall, although precision-cut liver slices (i.e., with regular and reproducible thickness) were obtained, the slice accuracy (here defined as the match between the nominal step size chosen and the actual slice thickness obtained after sectioning and equilibration) was poor. In particular, the actual slice thickness was found to be consistently higher than the nominal set step-size selected for slicing the tissue likely because the very floppy and compliant fresh hepatic tissue swells significantly during post-sectioning equilibration.

### Murine brain

#### PFA-fixed brain

**Figure 4:**
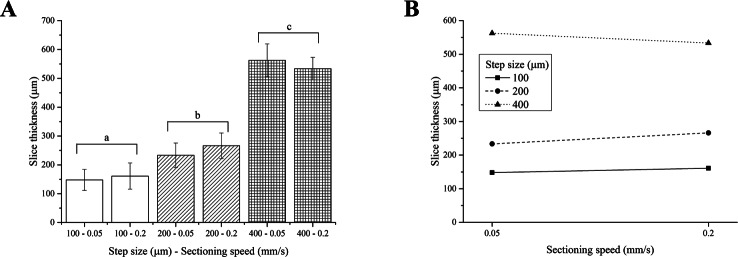
PFA-fixed brain slice thicknesses. (A) Bar plot: different letters indicate significant differences between samples (*p* < 0.05). Different bar fillings indicate different step sizes. (B) Two-way ANOVA interaction plot: the interaction between the step size and the sectioning speed is not significant. Error bars represent standard deviations.

Two-way ANOVA analysis indicates that the thickness of PFA-fixed brain slices depends on the step size only, yielding 3 different groups of slice thicknesses, regardless of the sectioning speed (Fig. 4A). As observed for fresh liver, the interaction between the step size and the sectioning speed was not significant (Fig. 4B).

Although again generally higher than nominal values, the thickness of PFA-fixed brain slices matched the vibratome step sizes better than those obtained for fresh liver. This tissue is in fact stiffer than the fresh liver, hence easier to accurately cut in thin regular and more precise slices and swells less than fresh liver during equilibration.

#### Fresh brain

**Figure 5:**
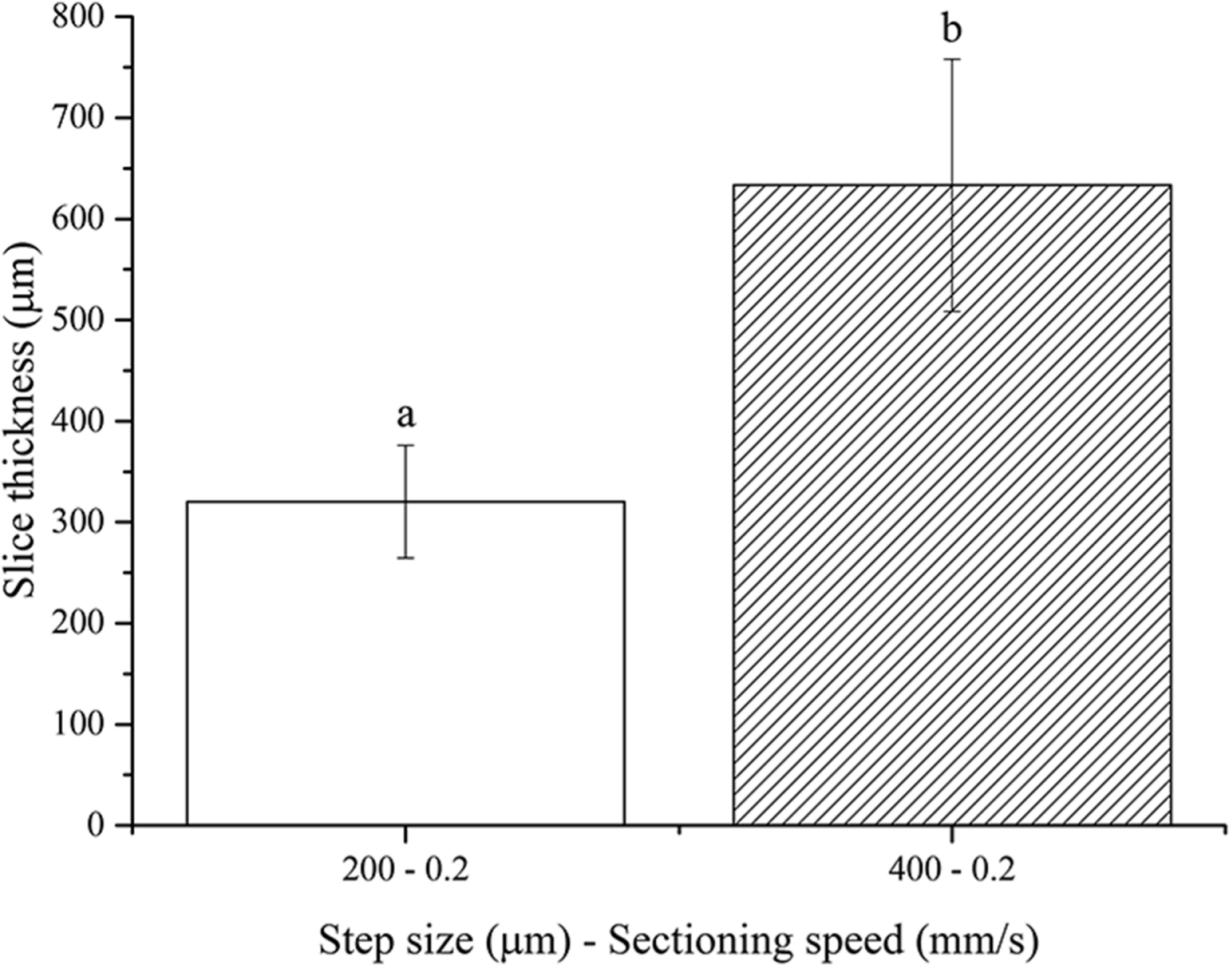
Fresh brain slice thicknesses. Bar plot: different letters indicate significant differences between samples (*p* < 0.05). Different bar fillings indicate different step sizes. Error bars represent standard deviations.

No slices were obtained using the 100 µm step size, once again because of the floppy and compliant nature of non-fixed soft tissues. One-way ANOVA analysis showed that the resultant thickness of fresh brain slices depends significantly on the step size chosen (Fig. 5). Again, the actual slice thicknesses were found to be higher than nominal vibratome step sizes. These results are in agreement with those obtained for both fresh pig liver and PFA-fixed murine brain, as expected since the stiffness of the non-fixed brain tissue is between those of the other two tissues investigated in this work.

## Conclusions

Precision-cut tissue slices from fresh porcine liver as well as from PFA-fixed and fresh murine brain were characterized in their thickness through a semi-automated slice analysis GUI implemented in Matlab^®^. Although precision-cut slices (i.e., with regular and repeatable thickness) were obtained for all the tissues investigated, the results presented in this work suggest that the match between the nominal step size chosen and the actual slice thickness obtained (i.e., slice accuracy) increases with tissue stiffness, from fresh liver to PFA-fixed brain. In particular, considering all the step size-sectioning speed combinations investigated for each tissue, the overall actual thickness change decreases from +100.8% for fresh pig liver, to +59.2% and +38.8% for non-fixed and PFA-fixed murine brain, respectively (Table 1). Since the block advance calibration tests show that the vibratome slicing is accurate to within 5%, the change in thickness is mainly due to swelling of slices during equilibration. In particular, the softer the tissue the higher the difference between the nominal step size and the actual thickness, as expected from the inverse relation between stiffness and swelling exhibited by most soft materials (Beamish et al., 2010; Cha et al., 2011). These results suggest that tissue stiffness, which increases from fresh pig liver to PFA-fixed murine brain tissue, plays a key role in determining the accuracy of vibratome-sliced sections. Both the oscillation amplitude and blade angle variables should be considered to complete the analysis of the effect of different vibratome sectioning parameters on the resultant slice thickness; however, they are likely to have a minor impact on the latter. Therefore, they were set to a constant value in the present work, in accordance with other reports (Zimmermann et al., 2009; Vaira et al., 2010).

**Table 1:** Slice thickness errors obtained for the different step size-sectioning speed combinations investigated. Errors are also reported as overall values for each tissue. The results show that slice accuracy increases from pig liver to fresh brain to fixed brain, suggesting that it is correlated with tissue stiffness.

	Slicing parameters		Actual thickness (μm)		Thickness error = (Mean value for actual thickness-step size)/step size	
	Step-size (μm)	Sectioning speed (mm/s)	Mean	Std. Dev.	(%)	Overall (%)
**Fresh pig liver**	100	0.1	–	–	–	100.8
	100	0.2	–	–	–	
	100	0.4	–	–	–	
	200	0.1	460.4	103.8	130.2	
	200	0.2	407.3	81.9	103.65	
	200	0.4	549.8	63.9	174.9	
	400	0.1	575.6	70.0	43.9	
	400	0.2	591.5	63.9	47.875	
	400	0.4	819.2	142.7	104.8	
**Fresh murine brain**	100	0.2	–	–	–	59.2
	200	0.2	320.3	55.7	60.15	
	400	0.2	633.4	124.6	58.35	
**PFA-fixed murine brain**	100	0.05	148	36.5	48	38.8
	100	0.2	161.1	45.2	61.1	
	200	0.05	233.2	42.5	16.6	
	200	0.2	266.2	44.5	33.1	
	400	0.05	562.6	56.9	40.65	
	400	0.2	533.5	39.1	33.375	

The agarose-embedding strategy used to characterize the actual slice thickness after vibratome sectioning and equilibration better preserves sample geometry in the fully hydrated state (i.e., that of tissue slices during *in vitro* cultures) with respect to the widely used paraffin embedding technique. The latter is likely to result in significant sample shrinkage during the inclusion process which inevitably leads to an underestimation of the slice thickness, and may explain why several papers report a reduction in thickness with respect to the nominal vibratome step size.

The method we report here enables the evaluation of slice thickness in the hydrated state typical of tissue culture experiments. Similar results cannot be obtained using classical techniques such as step advance calibration (which does not consider deformation or swelling after cutting) and paraffin embedding (which involves sample dehydration). In conclusion, given the potential benefits and advantages of precision-cut slices in many biological and biomedical engineering applications, the quantitative evaluation of the effects of the vibratome’s sectioning parameters on the actual thickness performed in this work can be very helpful for establishing the most suitable slicing setup for a given tissue.

## Additional Information and Declarations

### Data Deposition

The following information was supplied regarding the deposition of related data: The software Stegun can be downloaded at http://www.centropiaggio.unipi.it/software.
